# Supplementary material for: A ventrolateral medulla-midline thalamic circuit for hypoglycemic feeding
Source: Nat Commun. 2020 Dec 4;11:6218. doi: 10.1038/s41467-020-19980-7 (PMC7719163; doi:10.1038/s41467-020-19980-7)
Supplement: Supplementary file 1 — Supplementary Information [file 41467_2020_19980_MOESM1_ESM.pdf]

Supplementary Information

A ventrolateral medulla-midline thalamic circuit for hypoglycemic feeding

Beas, Gu et al

Contains: Supplementary Figures 1-11

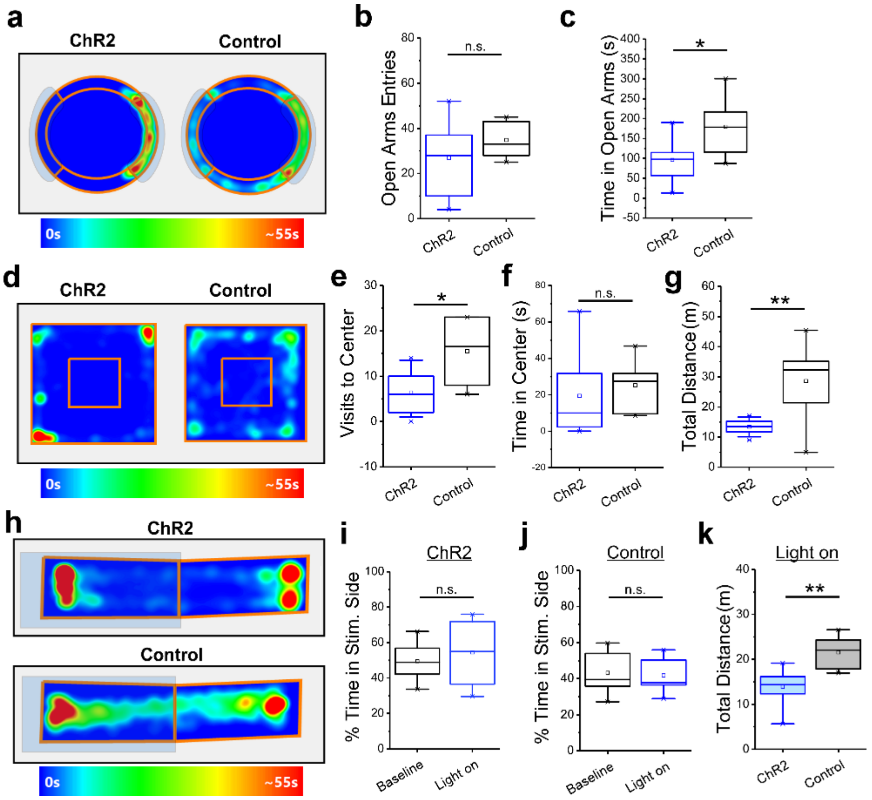

Supplemental Figure 1. Optogenetic stimulation of VLM^{CA} terminals in the pPVT promotes anxiogenic behavior and decreases locomotion. a. Representative heat maps of subject performance in the EZM for ChR2 or control virus expressing mice. Shaded areas represent closed arms. **b**. Quantification of open arm entries in the EZM. Control (black), 34.80 ± 3.98, n = 5 mice; ChR2 (blue), 26.89 ± 5.82, n = 9 mice; two-sided Unpaired t-test, P=0.37. **c**. Quantification of time spent in open arms. Control, 179.18 ± 30.95, n = 6 mice; ChR2, 95.93 ± 17.18, n = 9 mice; two-sided Unpaired t-test, *P=0.02. **d**. Representative heat maps of activity during the OFT for ChR2 and control virus expressing mice. **e**. Quantifications of visits to the center. Control, 15.50 ± 3.17, n = 6 mice; ChR2, 6.40 ± 1.55, n = 10 mice; two-sided Unpaired t-test, *P=0.011. **f**. Quantification of time spent in the center. Control, 25.17 ± 5.94, n = 6 mice; ChR2, 19.36 ± 6.67, n = 10 mice; two-sided Unpaired t-test, P=0.56. **g**. Quantification of total distance traveled. Control, 28.55 ± 5.71, n = 6 mice; ChR2, 13.42 ± 0.79, n = 10 mice; two-sided Unpaired t-test, *P=0.004. **h**. Representative heat maps of activity during the RTPP test for ChR2 and control virus expressing mice. Shaded areas represent the side of the chamber where stimulation was given. **i**. Quantification of percent time spent in the stimulated side before (Baseline) and after stimulation (Light on) for ChR2 expressing mice. Baseline, 49.45 ± 2.82; Light on, 54.44 ± 5.20, n = 12 mice; two-sided Paired t-test, P=0.30. **j**. Quantification of percent time spent in the stimulated side for Baseline and Light on periods for control mice. Baseline, 43.21 ± 5.98; Light on, 41.84 ± 4.93, n = 5 mice; two-sided Paired t-test, P=0.88. **k**. Quantification of total distance traveled during Light on for ChR2 and control virus expressing mice. Control (gray), 21.52 ± 1.84, n = 5 mice; ChR2 (blue), 13.89 ± 1.06, n = 12 mice; two-sided Unpaired t-test, **P=0.002. Box chart legend: box is defined by 25th, 75th percentiles, whiskers are determined by 5th and 95th percentiles, and mean is depicted by the square symbol. Data presented as mean ± SEM.

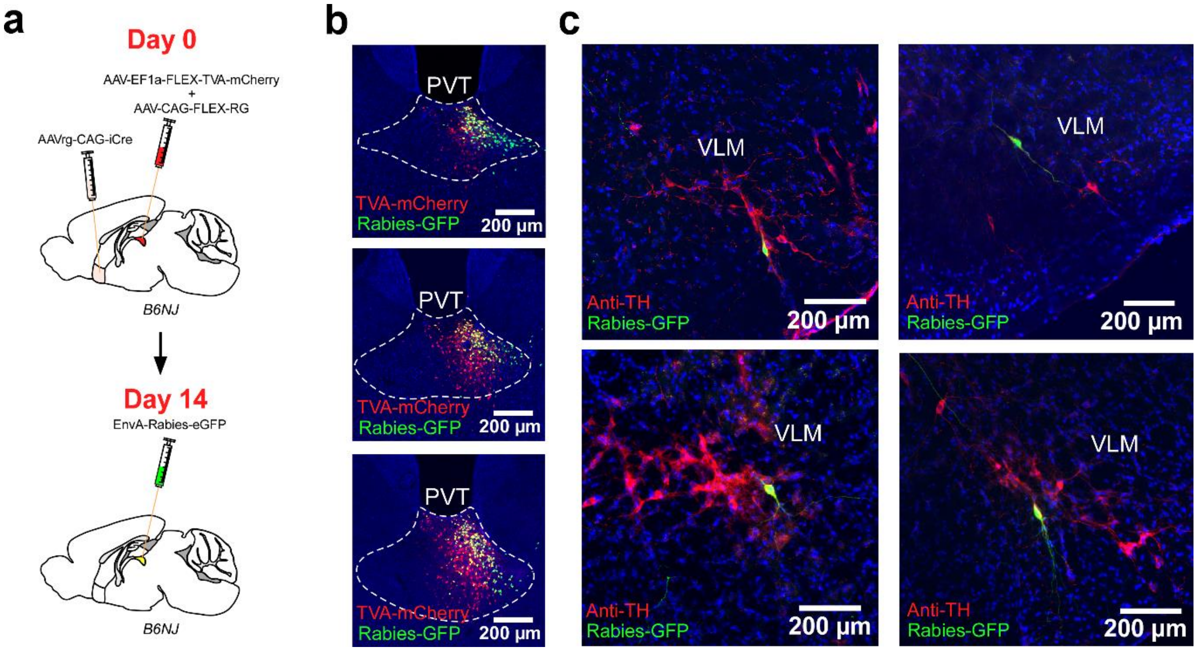

Supplementary Figure 2. Pseudo-typed rabies tracing of monosynaptic inputs onto NAc-projecting pPVT neurons. a. Schematic of the viral vector strategy employed for the pseudo-typed rabies tracing of monosynaptic inputs to pPVT–NAc neurons. **b**. Representative images of the pPVT from a sample subject showing the location of the rabies starter cells (Rabies-GFP and TVA-mCherry double-labelled cells). **c**. Representative images of Rabies-GFP (retrogradely-labelled) expression in the VLM region and show colocalization with TH immunostaining (Anti-TH; red). Data from 3 mice. This experiment was independently repeated twice with similar results.

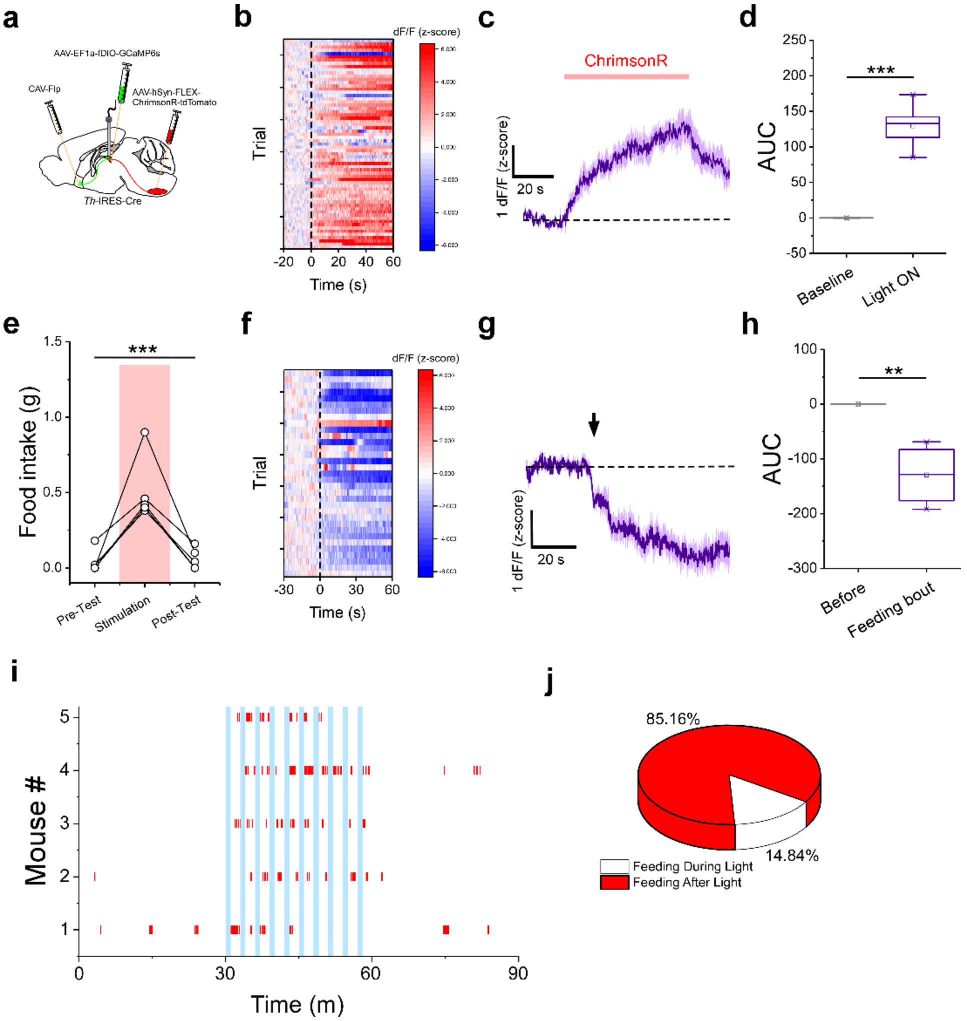

Supplementary Figure 3. An alternative viral approach that eliminates potential contamination of non-catecholaminergic neurons of the VLM confirms VLM^{CA}-mediated activation of pPVT–NAc neurons *in vivo*. a. Schematic of an alternative viral vector strategy used for selectively expressing GCaMP6s in NAc-projecting neurons of the pPVT of TH-Cre mice. Notice that GCaMP6s expression was achieved by bilateral injections of CAV-Flp into the NAc, and Flp-dependent GCaMP6s in the pPVT. **b**. Heatmap showing individual trial GCaMP6s responses to light stimulation (561 nm at 20 Hz). **c.** Average GCaMP6s responses from pPVT–NAc neurons upon light stimulation. **d.** Quantification of light-evoked changes in GCaMP6s fluorescence in pPVT–NAc neurons. Area under the curve (AUC), Baseline, -0.09 ± 0.20; Light ON, 129.22 ± 14.76; two-sided Paired t-test, ***P=0.0009. **e.** Quantification of food intake during VLM^{CA}-pPVT stimulation showing increases in feeding after stimulation in well-fed ChrimsonR expressing mice. Total food intake in grams, Pre-Test: 0.04 ± 0.04; Stimulation: 0.512 ± 0.10; Post-Test, 0.07 ± 0.03, n = 5 mice; $F(2,12) = 18.14$, one-way ANOVA followed by Tukey's test. Group comparisons: Pre-Test vs Stimulation, ***P=0.0005; Post-Test vs Stimulation, ***P=0.0007. **f.** Heatmap of individual trials GCaMP6s responses from pPVT–NAc neurons to the initiation of feeding bouts. **g.** Average GCaMP6s responses from pPVT–NAc neurons during feeding bouts. **h**. Quantification of feeding bouts onset-related changes in GCaMP6s fluorescence in pPVT–NAc neurons. AUC, Baseline, -0.10 ± 0.10; Feeding bout, -129.40 ± 28.30; n = 4 mice, two-sided Paired t-test, *P=0.02. **i**. Scatter plot shows feeding events for individual mice as they

occur during the stimulation protocol (1min ‘light on’ - blue lines, 2min ‘light off’ -white background). Notice that most of the feeding behavior occurred upon termination of light stimulation and during ‘light off’ periods. **j**. Pie chart summarizing the proportion of feedings that took place during (white) and after (red) light stimulation. Feeding during light: 14.84%; Feeding after light: 85.16%. n=5 mice. Box chart legend: box is defined by 25th, 75th percentiles, whiskers are determined by 5th and 95th percentiles, and mean is depicted by the square symbol. Data presented as mean ± SEM.

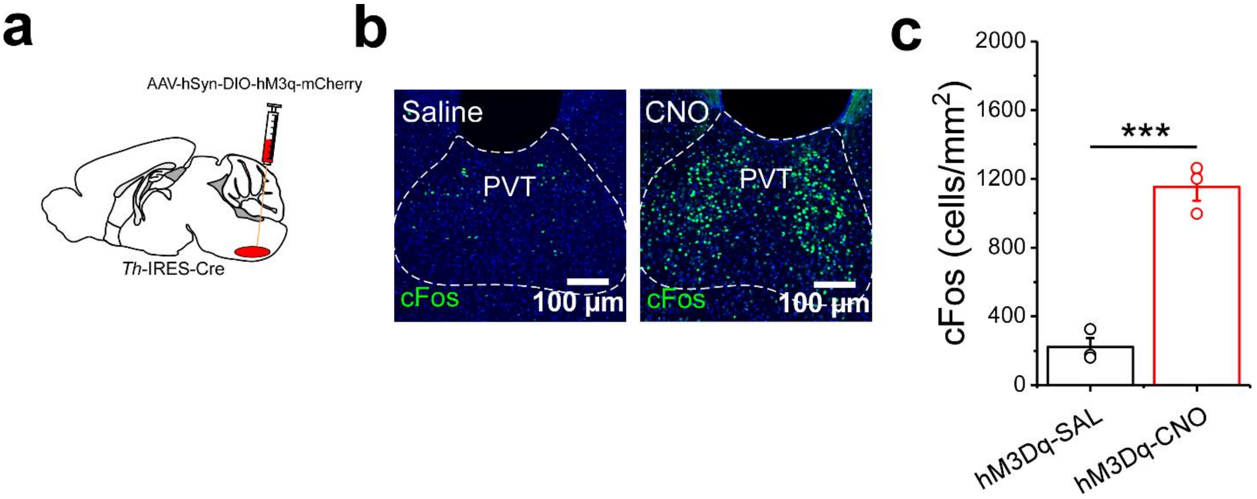

Supplementary Figure 4. Chemogenetic activation of VLM^{CA} neurons markedly increased the expression of the immediate early gene cFos in pPVT-neurons. a. Schematic of the stereotaxic injections for selective expression of the excitatory DREADD (hM3q) in VLM^{CA} of TH-IRES-Cre mice. **b**. Representative images showing cFos immunohistochemistry in the pPVT of these mice when injected with saline (left), or CNO to chemogenetically stimulate VLM^{CA} neurons (right). **c**. Quantification of cFos immunoreactivity in the PVT in cells/mm^2, hM3q-SAL, 220.08 ± 52.71, n = 3 mice; hM3q-CNO, 1153.04 ± 80.13, n = 3 mice; two-sided Unpaired t-test, ***P=0.0006. Data presented as mean ± SEM.

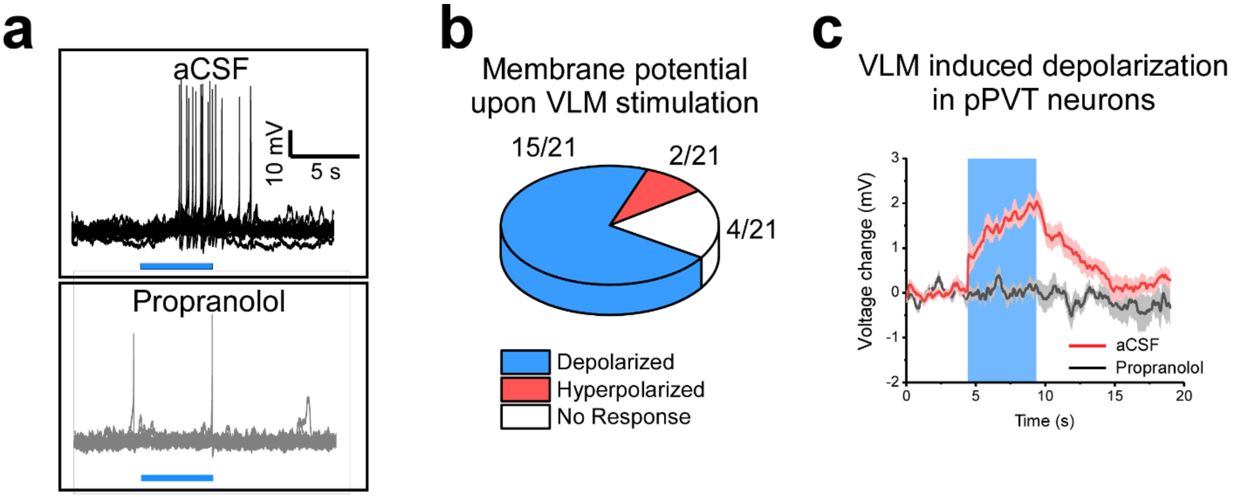

Supplemental Figure 5. Optogenetic stimulation of VLM^{CA} terminals induces adrenergic receptor-mediated depolarizations in pPVT neurons a. Sample traces showing the effect of VLM^{CA} axonal input photostimulation on the spontaneous firing of a pPVT neuron in the presence (bottom) and absence (top) of the adrenergic beta receptor blocker, propranolol. Optogenetic stimulation consisted of blue light pulses of 1 ms, delivered at 20 Hz for 5 s (see Methods). **b**. Pie chart summarizing the proportion of pPVT cells that showed depolarized (blue), hyperpolarized (red), and neutral (white) responses to photostimulation of ChR2-expressing VLM^{CA} inputs. Neurons were defined as depolarized or hyperpolarized by light stimulation if the membrane potential changed a minimum of 3 standard deviations from baseline. Depolarized: n = 15 neurons from 3 mice; Hyperpolarized: n = 2 neurons from 3 mice; No Response n = 4 neurons from 3 mice. **c**. Average plot depicting the effect of optogenetic stimulation of VLM^{CA} terminals expressing ChR2 on the membrane potential of pPVT neurons in the presence (n = 8 neurons, 3 mice) or absence (n = 15 neurons, 3 mice) of the adrenergic beta receptor blocker propranolol (30 µM). Data presented as mean ± SEM.

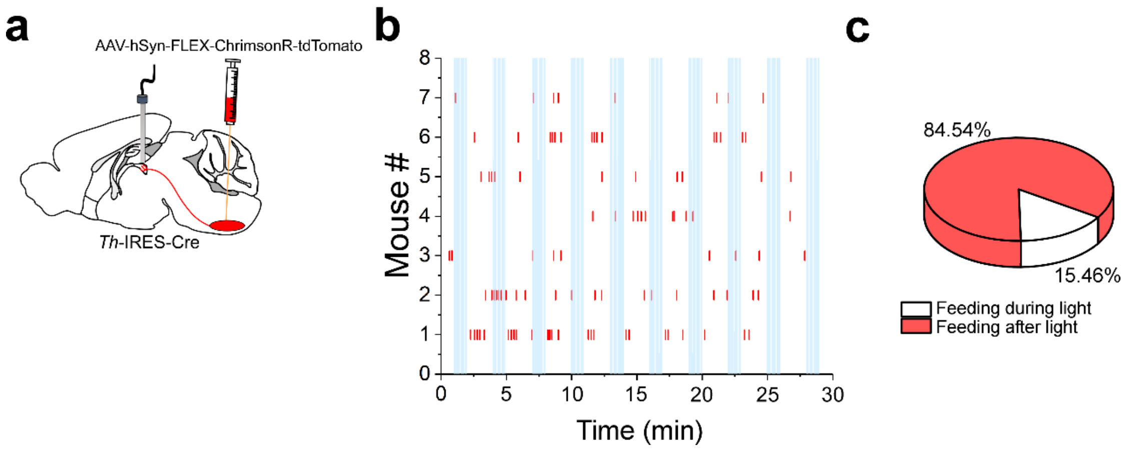

Supplementary Figure 6. Temporal distribution of feeding episodes during optogenetic stimulation of VLM^{CA}–pPVT projections. a. Schematic of the stereotaxic injections for selective expression of ChrimsonR-tdTomato in VLM^{CA} neurons of TH-IRES-Cre mice. **b**. Scatter plot shows feeding events for individual mice as they occur during the stimulation protocol (1min 'light on' - blue lines, 2min 'light off' -white background). Notice that most of the feeding behavior occurred upon termination of light stimulation and during 'light off' periods. **c**. Pie chart summarizing the proportion of feedings that took place during (white) and after (red) light stimulation. Feeding during light: 15.46%; Feeding after light: 84.54%. n= 7 mice.

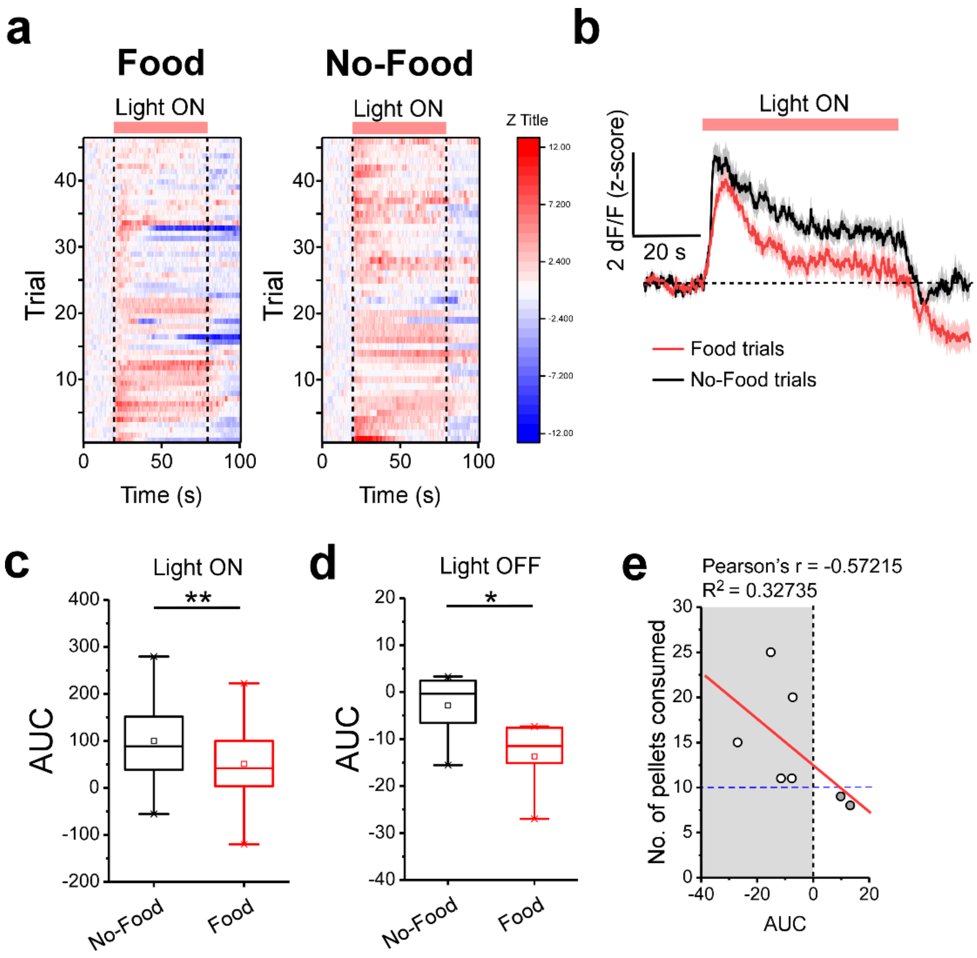

Supplementary Figure 7. Feeding decreases the activity of pPVT–NAc neurons after photostimulation of VLM^{CA} axonal inputs to pPVT. a. Left: Heatmap of GCaMP6s responses from pPVT–NAc neurons to light stimulation (561 nm at 20 Hz) in sessions were food was provided (Food), allowing mice to eat in response to light stimulation. Right: Heatmap of GCaMP6s responses from pPVT–NAc neurons to light stimulation (561 nm at 20 Hz) in sessions were food was not provided (No-Food) and thus mice were not able to eat in response to light stimulation. **b.** Comparison of average GCaMP6s responses from pPVT–NAc neurons upon light stimulation from Food and No-Food sessions. **c.** Quantification of light-evoked changes in GCaMP6s fluorescence in pPVT–NAc neurons comparing Food and No-Food sessions. Area under the curve (AUC), No-Food 99.53 ± 11.17; Food, 50.45 ± 10.73; two-sided Paired t-test, **P=0.002. **d.** Quantification of changes in GCaMP6s fluorescence in pPVT–NAc neurons after light stimulation comparing Food and No-Food sessions. Area under the curve (AUC), No-Food -2.87 ± 2.93; Food, -13.72 ± 3.62; two-sided Paired t-test, **P=0.04. **e.** Correlation between the quantification of decreases in GCaMP6s fluorescence in pPVT–NAc neurons after light stimulation (Light OFF), measured by area under the curve (AUC), and number of pellets consumed after photostimulation of VLM^{CA} axonal inputs to pPVT. No. of pellets consumed in the session was negatively correlated with AUC. Box chart legend: box is defined by 25th, 75th percentiles, whiskers are determined by 5th and 95th percentiles, and mean is depicted by the square symbol. Data presented as mean ± SEM.

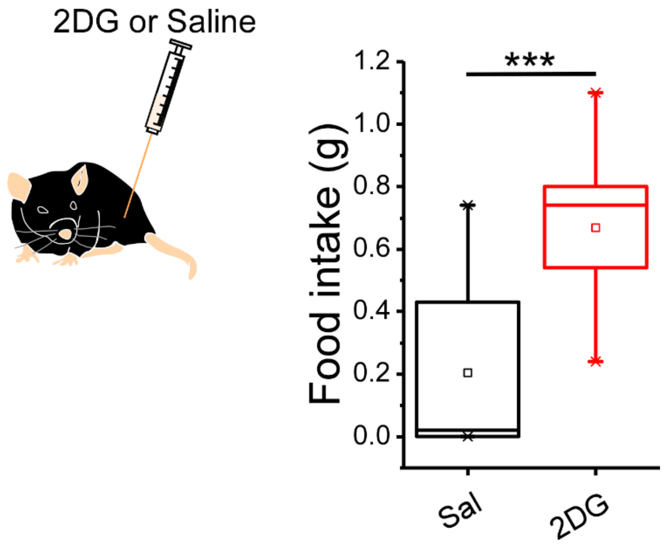

Supplementary Figure 8. Pilot experiment confirming that 2DG induces robust feeding in well-fed mice. Left: Well-fed mice were injected with either 2DG to induce glucoprivation or with saline as control. Right: Quantification of food intake for a 5 hr period after either saline or 2DG administration. Total food intake in grams, Sal, 0.20 ± 0.06, n = 20 mice; 2DG, 0.67 ± 0.06, n = 17 mice; two-sided Unpaired t-test, ***P=0.000004. This experiment was independently repeated three times with similar results. Box chart legend: box is defined by 25th, 75th percentiles, whiskers are determined by 5th and 95th percentiles, and mean is depicted by the square symbol. Data presented as mean ± SEM.

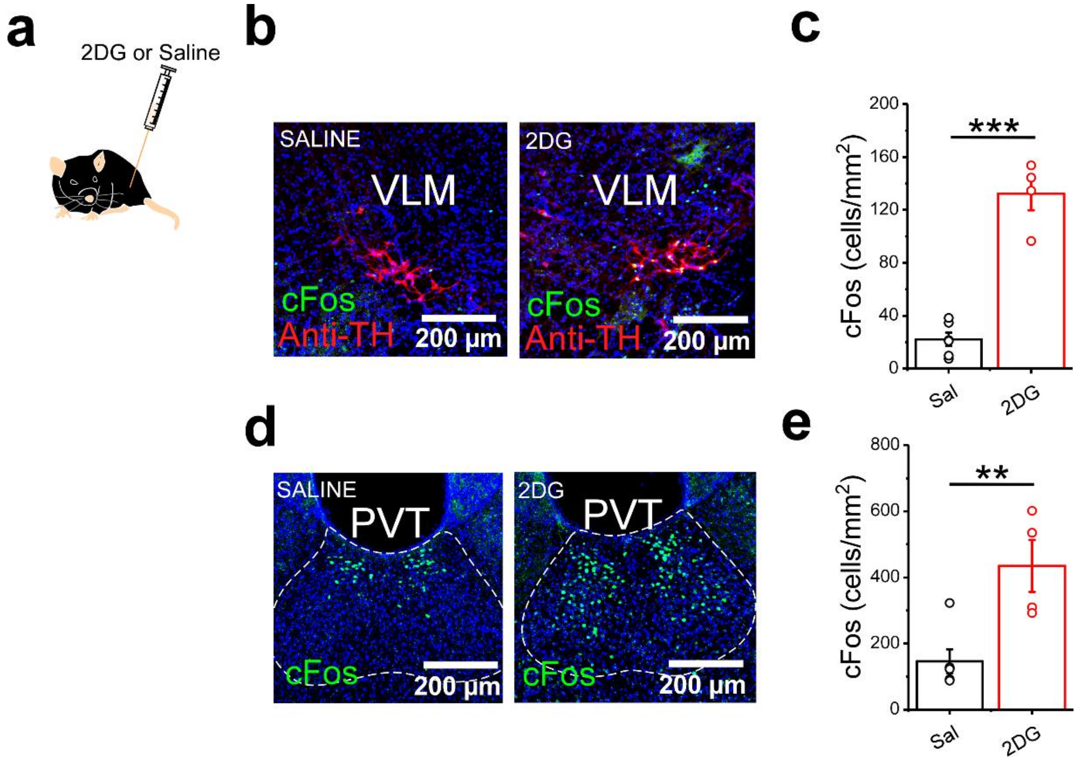

Supplementary Figure 9. 2DG increases the expression of the immediate early gene cFos in both VLM^{CA} and pPVT-neurons. a. Diagram of the experimental design used to induce glucoprivation. Mice were injected with either 2DG to induce glucoprivation or with saline as control. **b**. Representative images showing saline- (left) and 2DG-induced (right) cFos expression in VLM^{CA} neurons (depicted by their TH immunoreactivity). **c**. Quantification of cFos immunoreactivity in VLM^{CA} neurons shown as cells/mm^2, Sal, 22.04 ± 5.13, n = 6 mice; 2DG, 132.22 ± 12.56, n = 4 mice; two-sided Unpaired t-test, ***P=0.00001. **d**. Representative images showing saline- (left) and 2DG-induced (right) cFos expression in the pPVT. **e**. Quantification of c-Fos immunoreactivity in the pPVT shown as cells/mm^2, Sal, 145.86 ± 35.96, n = 6 mice; 2DG, 434.16 ± 78.37, n = 4 mice; two-sided Unpaired t-test, **P=0.005. This experiment was independently repeated twice with similar results. Data presented as mean ± SEM.

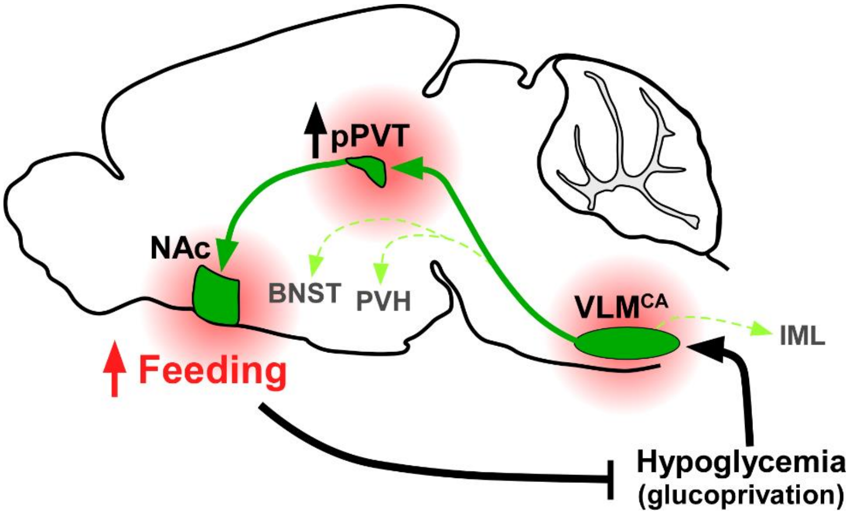

Supplementary Figure 10. Glucoprivation-induced feeding mediated by the VLM^{CA}–pPVT–NAc pathway. Summary model of the circuit that mediates glucoprivation-induced feeding behavior: the VLM^{CA}–pPVT–NAc pathway.

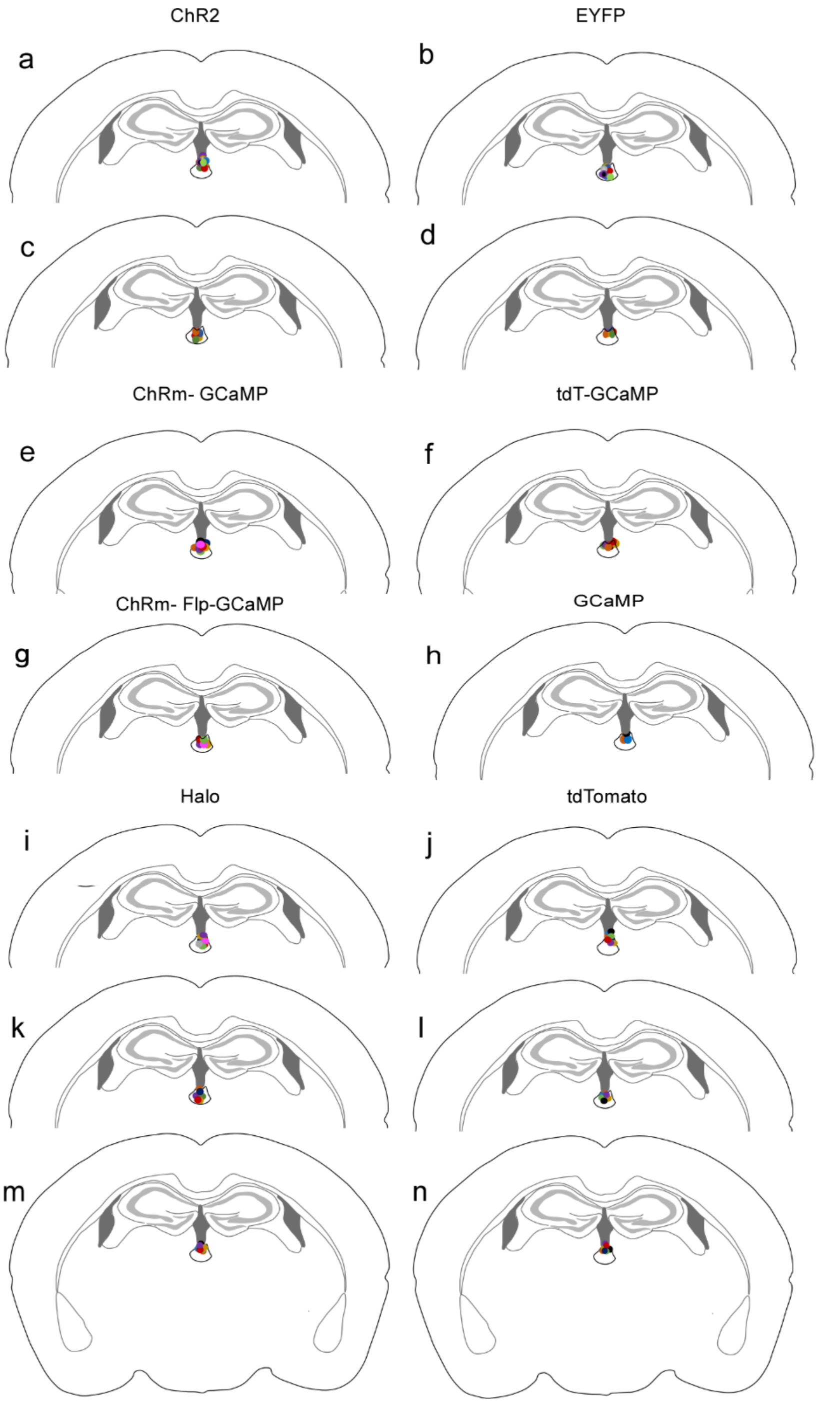

Supplementary Figure 11. Optical fiber placements. a. ChR2 group for the feeding experiment shown in Fig. 2d, e. **b.** Control subjects for the feeding experiment in Fig. 2f, g. **c.** ChR2-expressing mice used for the affective assays shown in Supplementary Fig. 1. **d.** Control mice used for the affective assays shown in Supplementary Fig. 1. **e.** ChrimsonR-expressing mice included in Fig. 3. **f.** tdTomato-expressing mice included in Fig. 3. **g.** ChrimsonR-expressing mice included in Supplementary Fig. 3. **h.** GCaMP6s-expressing mice included in Fig. 4. **i.** pPVT optical fiber placement for mice expressing halorhodopsin (eNpHR) in VLM^{CA} neurons for the 2DG-

evoked feeding experiment shown in Fig. 7c. **j.** pPVT optical fiber placement for mice expressing tdTomato in VLM^{CA} neurons for the 2DG-evoked feeding experiment shown in Fig. 7c. **k.** halorhodopsin mice used for the food restriction experiment shown in Fig. 7f. **l.** tdTomato mice used for the food restriction experiment shown in Fig. 7f. **m.** halorhodopsin mice used for the CORT and glucose measurements in 2DG-injected mice included in Fig. 7d, e. **n.** tdTomato mice used for the CORT and glucose measurements in 2DG-injected mice included in Fig. 7d, e. All circles depict the lowest position of the optical fibers for each subject.
