# Supplementary material for: A ventrolateral medulla-midline thalamic circuit for hypoglycemic feeding
Source: Nat Commun. 2020 Dec 4;11:6218. doi: 10.1038/s41467-020-19980-7 (PMC7719163; doi:10.1038/s41467-020-19980-7)
Supplement: Supplementary file 3 — Description of Additional Supplementary Files [file 41467_2020_19980_MOESM3_ESM.pdf]

Description of Additional Supplementary Files

File Name: Supplementary Movie 1

Description: **Feeding behavior induced by optogenetic stimulation of VLM^{CA} terminals in the pPVT.** Representative movie illustrating the feeding phenotype associated with optogenetic stimulation of the VLM^{CA}–pPVT pathway. The first two minutes of the video illustrate typical behavior during the 30 min pre-Test. The remainder of the video shows behavior associated with light simulation of VLM^{CA}–pPVT terminals. Three consecutive sample trials are included. Notice that in contrast to behavior displayed in the pre-Test, optogenetic stimulation is associated with decreased locomotion (as presented in Supplementary Fig. 1), followed by robust feeding behavior upon cessation of light stimulation (as presented in Supplementary Fig. 3i, j and 6). The food dispenser is located in the bottom right corner of the video.
