# Supplementary material for: A ventrolateral medulla-midline thalamic circuit for hypoglycemic feeding
Source: Nat Commun. 2020 Dec 4;11:6218. doi: 10.1038/s41467-020-19980-7 (PMC7719163; doi:10.1038/s41467-020-19980-7)
Supplement: Supplementary file 5 — Reporting Summary [file 41467_2020_19980_MOESM5_ESM.pdf]

nature research

Corresponding author(s): Mario A. Penzo

Last updated by author(s): Nov 4, 2020

Reporting Summary

Nature Research wishes to improve the reproducibility of the work that we publish. This form provides structure for consistency and transparency in reporting. For further information on Nature Research policies, see our Editorial Policies and the Editorial Policy Checklist.

Statistics

For all statistical analyses, confirm that the following items are present in the figure legend, table legend, main text, or Methods section.

n/a	Confirmed	
☐	☒	The exact sample size (n) for each experimental group/condition, given as a discrete number and unit of measurement
☐	☒	A statement on whether measurements were taken from distinct samples or whether the same sample was measured repeatedly
☐	☒	The statistical test(s) used AND whether they are one- or two-sided<br>*Only common tests should be described solely by name; describe more complex techniques in the Methods section.*
☒	☐	A description of all covariates tested
☐	☒	A description of any assumptions or corrections, such as tests of normality and adjustment for multiple comparisons
☐	☒	A full description of the statistical parameters including central tendency (e.g. means) or other basic estimates (e.g. regression coefficient) AND variation (e.g. standard deviation) or associated estimates of uncertainty (e.g. confidence intervals)
☐	☒	For null hypothesis testing, the test statistic (e.g. F, t, r) with confidence intervals, effect sizes, degrees of freedom and P value noted<br>*Give P values as exact values whenever suitable.*
☒	☐	For Bayesian analysis, information on the choice of priors and Markov chain Monte Carlo settings
☒	☐	For hierarchical and complex designs, identification of the appropriate level for tests and full reporting of outcomes
☒	☐	Estimates of effect sizes (e.g. Cohen's d, Pearson's r), indicating how they were calculated

Our web collection on statistics for biologists contains articles on many of the points above.

Software and code

Policy information about availability of computer code

Data collection	Fiber photometry data and TTL-synced feeding bouts were recorded using Synapse version 92 (Tucker-Davis Technologies). Experimental schedule and data acquisition for the reward foraging task were accomplished using ABET II (Lafayette Instruments Neuroscience). Data from patch clamp whole-cell recordings were collected with pCLAMP 10 (Molecular Devices). Confocal images were obtained in ZEN (version 2.3, Carl Zeiss Microscopy, LLC).
Data analysis	All data were analyzed using Origin Pro 2016 (OriginLab Corp.), image analysis and cell counting were performed using ImageJ software (Fiji, version 2017 May 30), video tracking data from the foraging task was analyzed using Ethovision XT 12 (Noldus), and video tracking for affective behaviors was completed using ANY-maze version 5. Data from patch clamp whole-cell recordings were analyzed using Calmpfit (pCLAMP 10 suite, Molecular Devices).

For manuscripts utilizing custom algorithms or software that are central to the research but not yet described in published literature, software must be made available to editors and reviewers. We strongly encourage code deposition in a community repository (e.g. GitHub). See the Nature Research guidelines for submitting code & software for further information.

Data

Policy information about availability of data

All manuscripts must include a data availability statement. This statement should provide the following information, where applicable:
- Accession codes, unique identifiers, or web links for publicly available datasets
- A list of figures that have associated raw data
- A description of any restrictions on data availability

All the data that support the findings presented in this study are available from the corresponding author upon reasonable request. Source Data are provided with this paper.

Field-specific reporting

Please select the one below that is the best fit for your research. If you are not sure, read the appropriate sections before making your selection.

- [x] Life sciences
- [ ] Behavioural & social sciences
- [ ] Ecological, evolutionary & environmental sciences

For a reference copy of the document with all sections, see nature.com/documents/nr-reporting-summary-flat.pdf

Life sciences study design

All studies must disclose on these points even when the disclosure is negative.

Sample size	The sample sizes used in our study, such as the numbers of neurons or animals, are about the same or exceed those estimated by power analysis (power = 0.8, α = 0.05).
Data exclusions	Mice without correct targeting of optical fibers, tracers and/or vectors were excluded from this study.
Replication	To ensure that our experimental findings can be reliably replicated, each experiment was repeated at least twice. Imaging and behavioral experiments where carried out in individual cohorts that were tested on separate occasions. Importantly, most experiments included data generated by multiple experimenters. All attempts of replicating the data were successful.
Randomization	For electrophysiological analyses, the sample size is 8-15 cells. For optogenetic-only feeding experiments, the sample size is 6-12 mice. For combined optogenetic and fiber photometry experiments, the sample size is 5-9 mice. For fiber photometry experiment in the reward foraging task, the sample size is 5 mice. For optogenetic experiments used to assess aversive and anxiety-like behavior, the sample size is 5-12 mice. For glucose and CORT assays, the sample size was 4-10 mice. For immunohistochemical cFos analyses, the sample size is 3-6 mice. All subjects were randomly allocated to the different experimental conditions.
Blinding	For all experiments in this study, subjects were randomly assigned to the various experimental conditions. However, the experimenters were not blinded to group allocation during data acquisition since the same experimenter had to administer the various treatments (e.g. saline, 2DG). Blinding was used for the analysis of feeding data for 2DG experiments (including those using chemogenetics). In addition, blinding was used for histological assessments including image analysis and cell counting.

Reporting for specific materials, systems and methods

We require information from authors about some types of materials, experimental systems and methods used in many studies. Here, indicate whether each material, system or method listed is relevant to your study. If you are not sure if a list item applies to your research, read the appropriate section before selecting a response.

Materials & experimental systems

n/a	Involved in the study	
	x	Antibodies
x		Eukaryotic cell lines
x		Palaeontology and archaeology
	x	Animals and other organisms
x		Human research participants
x		Clinical data
x		Dual use research of concern

Methods

n/a	Involved in the study	
x		ChIP-seq
x		Flow cytometry
x		MRI-based neuroimaging

Antibodies

Antibodies used	The primary antibodies: anti-cFos (1:1000, rabbit polyclonal, Millipore, catalog# ABE457; 1:50, rabbit monoclonal, Cell Signaling, catalog# 2250), validated for application (ABE457 and 2250) and specie (2250); and anti-TH (1:1000, chicken polyclonal, Aves Labs, catalog# AB_10013440), validated for application and specie. Fluorophore-conjugated secondary antibodies (conjugated with goat anti-rabbit Alexa Fluor-488 (A-11008) and goat anti-chicken Alexa Fluor-647 (A-21449)) were purchased from ThermoFisher Scientific and used at a 1:500 dilution. Antibodies were diluted in PBS with 10% NGS and PBST. For additional information on the validation of the antibodies used, please visit the manufacturer's website.
Validation	The anti-cFos and anti-TH antibodies were validated for both species and application by the manufacturer (please visit the company's website for details).

Animals and other organisms

Policy information about studies involving animals; ARRIVE guidelines recommended for reporting animal research

Laboratory animals	The following mouse line was used: TH-IRES-Cre mice (European Mouse Mutant Archive; stock number: EM:00254; backcrossed 5 generations with C57Bl/6NJ mice). In addition, we used C57BL/6NJ strain mice (The Jackson Laboratory). Both male and female mice 7–12 weeks of age were used for all experiments. Mice were housed under a 12-h light-dark cycle (6 a.m. to 6 p.m. light) at temperature of 70-74 °F and 40-65% humidity, with food and water available ad libitum.
Wild animals	No wild animals were used in this study.
Field-collected samples	No field-collected samples were used in this study.
Ethics oversight	All procedures were approved by the National Institute of Mental Health Animal Care and Use Committees performed following the Guide for the Care and Use of Laboratory Animals.

Note that full information on the approval of the study protocol must also be provided in the manuscript.
